# Experimental demonstration of novel beam characterization using a polarizable X-band transverse deflection structure

**DOI:** 10.1038/s41598-021-82687-2

**Published:** 2021-02-11

**Authors:** B. Marchetti, A. Grudiev, P. Craievich, R. Assmann, H.-H. Braun, N. Catalan Lasheras, F. Christie, R. D’Arcy, R. Fortunati, R. Ganter, P. González Caminal, M. Hoffmann, M. Huening, S. M. Jaster-Merz, R. Jonas, F. Marcellini, D. Marx, G. McMonagle, J. Osterhoff, M. Pedrozzi, E. Prat Costa, S. Reiche, M. Reukauff, S. Schreiber, G. Tews, M. Vogt, S. Wesch, W. Wuensch

**Affiliations:** 1 Deutsches Elektronen-Synchrotron, 22607 Hamburg, Germany; 2 CERN, 1211 Geneva 23, Switzerland; 3 PSI, 5232 Villigen, Switzerland; 4 Present Address: European XFEL GmbH, Holzkoppel 4, 22869 Schenefeld, Germany; 5 Present Address: Brookhaven National Laboratory, Upton, NY 11973-5000 USA

**Keywords:** Applied physics, Particle physics, Plasma physics

## Abstract

The PolariX TDS (Polarizable X-Band Transverse Deflection Structure) is an innovative TDS-design operating in the X-band frequency-range. The design gives full control of the streaking plane, which can be tuned in order to characterize the projections of the beam distribution onto arbitrary transverse axes. This novel feature opens up new opportunities for detailed characterization of the electron beam. In this paper we present first measurements of the Polarix TDS at the FLASHForward beamline at DESY, including three-dimensional reconstruction of the charge-density distribution of the bunch and slice emittance measurements in both transverse directions. The experimental results open the path toward novel and more extensive beam characterization in the direction of multi-dimensional-beam-phase-space reconstruction.

## Introduction

The characterization of slice properties of electron bunches, such as slice emittance, slice-energy spread and beam-current profile, is very useful in a variety of contexts.

In Free-Electron Lasers (FELs)^1^, those parameters are crucial for the optimization of the radiation emission process in the undulators. In the FEL community the beam is typically characterized by looking at the features of longitudinal slices having length equal to the cooperation length of the lasing mechanism.

In beam-driven Plasma Wake-Field Accelerators (PWFA), the longitudinal characterization of the beams driving the acceleration process is key for the achievement of high-quality acceleration. The control of the peak current profile of the driving beams is critical to optimize the efficiency of energy transfer in the system due to beam loading of the wakefields^2^. Also, due to the incredibly high accelerating and focusing gradients inherent to novel accelerator schemes the fidelity of the acceleration process is extremely sensitive to particular bunch properties. For example, offsets in the centroids of the beam in the transverse planes can drive strong instabilities during the acceleration process^3^ leading to beam losses.

Emittance^4^ is a property used to characterize the quality of a particle beam in terms of its size and divergence. It represents - or it is linked to - the volume occupied by the particles in their phase-space. Typically the characterization of the emittance and envelope of the beam in linear accelerators neglects the coupling terms in the transverse plane defined by the axis $x-y$. The measurement of the correlation terms in the beam covariance matrix^4^ is either extremely time-consuming, requiring very special machine optics^5^, and/or needs masks which can be realized only for low energy beams. Often only the correlation terms of the projected beam distribution are characterized^6–8^, not having access to the properties of the longitudinal slices of the beam.

We define longitudinal slice of the beam as a subset of particles around a specific longitudinal coordinate inside the bunch. Its definition comes from the need to average beam properties over a certain range of the longitudinal coordinate in order to achieve meaningful averages over statistically significant sub-ensembles.

Transverse deflection structures (TDS) allow longitudinal slice beam properties to be measured and are suitable for the detailed characterization of the phase-space of the bunch at critical locations of the beamline^9,10^.

The positioning of a TDS at the exit of the undulator section of the FEL at LCLS represented a game changer in the field of single-shot bunch characterization. It enabled not only the optimization of a number of different schemes for FEL lasing^11–17^ but also the documentation of the characteristics of each laser pulse delivered to the users^18^.

The development of a TDS working in the X-band frequency range was initiated at SLAC^19,20^ and then employed by many other facilities^21,22^. Due to the higher RF-frequency, X-band TDS have proven to be capable of achieving fs or sub-fs longitudinal resolution.

In 2016, a novel TDS-design, called PolariX TDS, was proposed at CERN, which provides flexibility in tuning the streaking direction of the electromagnetic field^23^.

**Figure 1:**
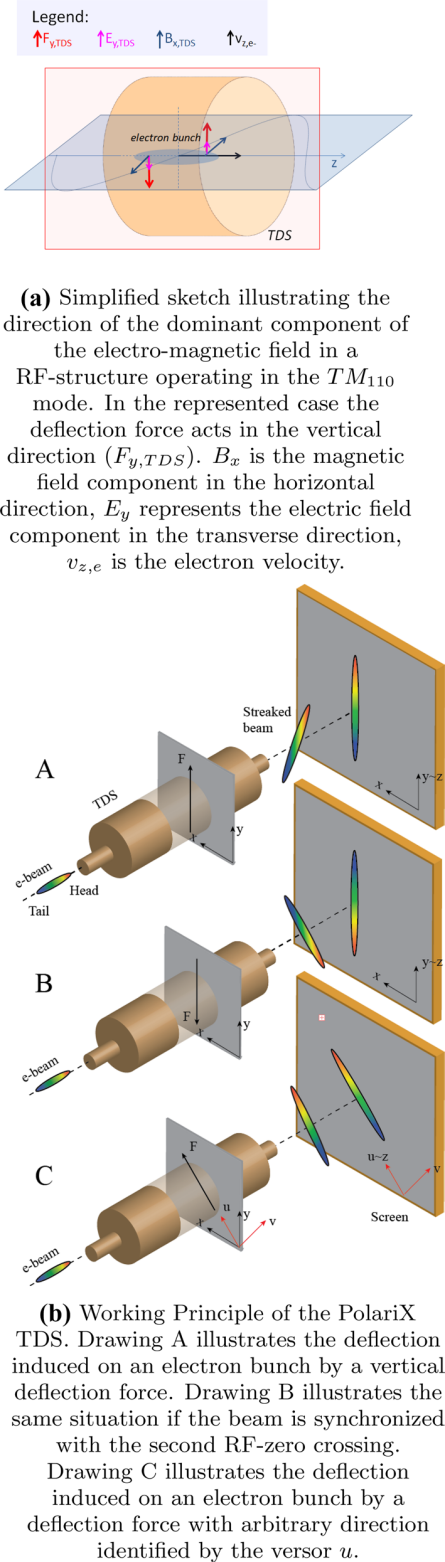
The PolariX TDS.

In Fig. 1 the working principle of the PolariX TDS is shown.

TDSs deflect electrons along a well-defined direction, which is perpendicular to the velocity of the electrons. When an electron bunch travels through such a device, each electron receives a kick, for example in the vertical direction, which is proportional to its distance from the center of the bunch. By taking a picture of the transverse distribution of the bunch visualized on a screen downstream of the TDS, it is possible to measure the properties of thin longitudinal slices of the electron bunch in the direction perpendicular to the one along which they have been streaked.

The PolariX TDS supports the propagation of both $TM_{110}$ (Transverse Magnetic) dipole-like modes. The superposition of those two modes defines the direction of the final streaking force. It is outside the scope of this article to give a description of the RF-design of the structure and its RF-network but we defer this description to references^23,24^. Here we give a simplified description of the fields in the structure which is accessible to a broader community of non-experts.

A sketch of the direction of the fields in the PolariX TDS for the case corresponding to a streaking in the vertical direction is shown in Fig.1a. The deflecting force $F_{y,TDS}$ experienced by the electron bunch is mainly due to the contribution of the magnetic field $B_{x,TDS}$ but also the transverse component electric field $E_{y,TDS}$ contributes.

According to on which zero of the sinusoidal field in the structure the beam is synchronized, the head of the beam will be deflected onto the upper part of the screen (as illustrated in Fig. 1b(A)) or onto the lower part of the screen (as illustrated in Fig. 1b (B)). The innovative feature of the PolariX TDS is the possibility of changing “on-line" the direction of the streaking field by controlling a phase-shifter located in the waveguide network. This results in the possibility of deflecting the electron bunch along an arbitrary direction, as illustrated in Fig. 1b(C). When the position of the phase-shifter is changed, the direction of the fields depicted in Fig.1a rotates about the z axis by a given angle.

We can define an equivalent transverse deflection voltage V that is linked to the deflecting force $F_u$ acting along the direction *u*, by the expression:

1 $$\begin{aligned} V=\frac{1}{e} \int _{0}^{L} F_u dz \end{aligned}$$

where *e* is the electron charge, *L* is the length of the RF-structure and *z* is the longitudinal spacial coordinate. For the PolariX TDS this voltage can be related to the input power $P_i$ using the expression^25^:

2 $$\begin{aligned} V[MV]=5.225 \cdot \sqrt{P_i[MW]}. \end{aligned}$$

As we will explain in the next section, high transverse deflection voltages are necessary to achieve sub-fs temporal resolution. The PolariX TDS has been designed and tested to stand input power up to more than 25 MW^25^. Nevertheless in this article we will present the first test done with an electron beam that has been performed using an RF-power source limited to 6MW peak-power.

The possibility of rotating the deflection field in the PolariX TDS opens up brand new opportunities for a broader characterization of electron beam and potentially can revolutionize the field of beam phase space characterization. We gain the possibility of characterizing the beam properties along an arbitrary axis *v* which is perpendicular to the streaking direction *u*, also defined in Fig. 1b(C). More specifically we can:

- measure beam slice properties, such as trajectory, transverse size, emittance, in both transverse planes, which we have explained to be fundamental for FEL performances and for the optimization of PWFA;
- perform three-dimensional charge-density measurement^26–28^, including quantitative evaluation of the offset of the centroids of the slices and measurement of their x-y correlation. This capability allows the detection of “skewed" perturbations caused by e.g. high order modes couplers^29^ or the control of sophisticated beam optics^30–32^;
- if the diagnostic beamline in which the TDS is installed can generate dispersion in both transverse planes, we have access to a full six-dimensional phase-space matrix.

In this article we focus in particular on the possibility of measuring the slice emittance of the beam in both the horizontal and vertical axis using the same RF-device and the first experimental three-dimensional reconstruction of the charge-density distribution of the beam using a tomographic algorithm. As we show, the latter measurement is conceptually simple and relatively quick, such that it can be incorporated in the fundamental characterization methods used for the optimization of a linac, giving access to the x-y correlation terms in the beam distribution for potentially countless applications.

The work that we describe in this article has been carried out within the PolariX TDS project, which aims at the prototyping and series production of the PolariX TDS for application in fs beam diagnostics. This project includes three institutes (DESY, CERN and PSI) and four experiments: FLASHForward, FLASH2 and SINBAD at DESY and the ATHOS beamline at SwissFEL, PSI^25,33,34^. The details of the RF-design of the prototype structure have been recently presented in^24^. In the same article the RF-field-properties and the high gradient performances of the structure are experimentally validated for the first time by using RF-field characterization methods.

## Results

### FLASHForward beamline

The FLASHForward experimental facility^35^ is a test bed for PWFA research^36^. The experiment occupies a third beamline of the FLASH FEL user facility^37^, sharing the same front end as the FEL users. The electron bunch is generated by an RF-photoinjector and initial acceleration is carried out by the FLASH superconducting radio-frequency (SRF) stage using superconducting niobium accelerating cavities. This, along with the FEL-quality feedback systems, generates high-quality, low-emittance, GeV-level electron bunches to the FLASHForward beamline.

The presence of a third-harmonic accelerating cavity in the FLASH linac provides immense flexibility over the first, second, and third derivatives of the longitudinal energy profile of the bunch. In connection with the bunch compression chicanes, the longitudinal current profile of the bunch can be manipulated. In addition to this, the FLASH facility is host to an S-band LOLA-type TDS^9,38^, routinely used to characterize the longitudinal bunch properties for FEL users^39,40^.

**Figure 2:**
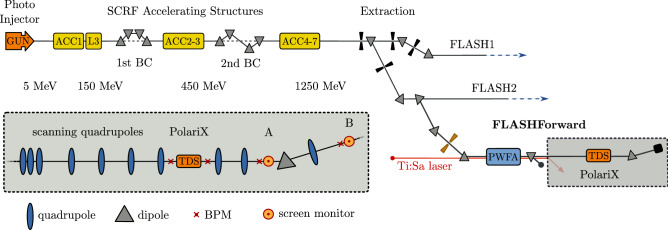
FLASH beamline layout with the three coexisting beamlines: FLASH1, FLASH2 and FLASHForward. A schematic layout of the PolariX-TDS diagnostics beamline is shown inside the dashed box with gray background. The quadrupoles upstream of the TDS are used to scan the phase advance in the slice emittance measurement. Screen stations marked with A and B correspond to 11FLFXTDS (slice emittance screen) and 8FLFDUMP (longitudinal phase space screen) respectively.

The FLASHForward facility was extended in the summer of 2019 to incorporate the PolariX TDS and its associated beamline components (see Fig. 2).

To stress the variable polarization feature of the PolariX TDS, in the following expressions *u* will designate the transverse direction parallel to the streaking force while *v* will designate the transverse direction perpendicular to *u*.

In order to maximize the temporal resolution of a transverse deflection system the optics of the beamline is typically set-up to minimize the longitudinal resolution parameter:

3 $$\begin{aligned} R_z = \frac{\sigma _u}{S} = \sqrt{\frac{\varepsilon _{u}(s)}{\beta _{u}(s_0)}} \frac{1}{| \sin {\mu _u}|} \frac{E}{eVk} \quad , \end{aligned}$$

where *S* is the streak parameter defined as:

4 $$\begin{aligned} S = \sqrt{\beta _{u}(s)\beta _{u}(s_0)} |\sin {\mu _u}(s_0,s)| \frac{eVk}{E} \quad , \end{aligned}$$

$\beta _{u}(s)$ and $\beta _{u}(s_0)$ are the betatron functions in the *u*-plane at the imaging screen and TDS, respectively, $\mu _u(s,s_0)=\int _{s_0}^{s}\frac{1}{\beta _u(s)}ds$ is the phase advance in the *u*-plane between the TDS and the screen, *E* is the beam energy, *V* is the cavity voltage, and *k* is the RF wave-number. The temporal resolution $R_t$ is defined as $R_z/c$, where *c* is the light velocity.

From an optics standpoint, a maximal streak requires a phase advance of $n\pi /2$ (where *n* is an odd integer) and large betatron function in the streaking plane at the TDS.

The betatron function in the TDS typically varies between 15 m and 50 m. The limit for the maximum applicable betatron function is set by the aperture of the structure, which has 4mm radius. There are several disadvantages in using a TDS with a small aperture including a tighter limit on the electron or photon beam transmission and stronger longitudinal and transverse wakefields acting on the particle beam. Those limitations have been studied in^24^ and judged acceptable.

For 6 MW peak input power in the PolariX structure a deflection voltage of almost 13 MV is expected^34^, which allows achieving sub-10 fs resolution for the highest values of betatron function in the TDS. Sub-10 fs resolution has been achieved during longitudinal phase-space measurements performed during the commissioning of the prototype structure, and the results are going to be presented in a separate publication^41^. In this article we will focus on the first experimental realization of slice emittance measurements on two transverse planes using the same RF-structure and three-dimensional beam charge density reconstruction. In those first demonstration-measurements we have consciously not yet pushed the temporal resolution to its best value, but we leave this optimization for future studies.

### Experimental verification of beam streaking at multiple directions

The commissioning of the PolariX TDS at FLASHForward started in September 2019. The cavity was operated with input power up to 6 MW.

The first feature which was experimentally validated during the commissioning of the structure with an electron bunch is the capability of the structure to streak the electrons at multiple directions. The change of the direction of the streaking is possible thanks to the remote adjustment of a phase-shifter which is located in the waveguide network.

**Figure 3:**
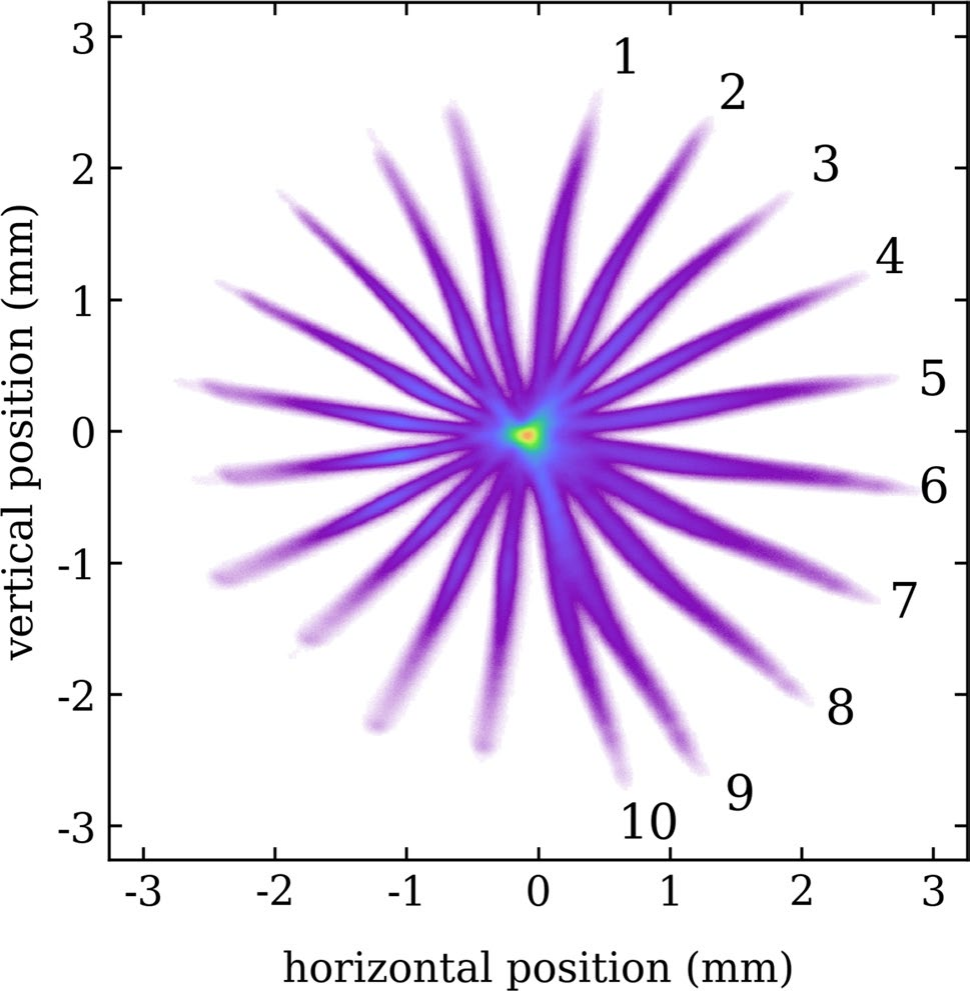
Streaked beam at multiple angles. The figure shows a composition where 10 pictures of streaked beam visualized at 11FLFXTDS have been combined. Each image has been taken for a different set-point of the RF phase shifter, as shown in Supplementary Movie 1. The profiles have been artificially centered and overlapped taking as reference the center of charge of the distributions for a better visualization of the variable angle of streaking.

Figure 3 shows some examples of beam distributions collected at 11FLFXTDS for different set-points of the phase-shifter, corresponding to different directions of the streaking field. The calibration of the streak parameter and the streak angle can be done simultaneously by scanning the position of the beam on the screen as a function of the RF-phase. During the scan, the centre of charge of the beam on the measurement-screen moves transversely along the streaking direction of the structure. The angle of the direction of the streaking can therefore be calculated by fitting the direction of the shift of the beam centroid, while the streak parameter can be calculated by measuring the amplitude of the shift for a given change in the RF-phase.

### Measurements of the horizontal and vertical slice emittance

As the PolariX TDS offers the possibility to deflect the beam into arbitrary directions, slice emittance measurements in both transverse directions can be performed using the same structure for both measurements.

To demonstrate this approach, dedicated measurements were performed at FLASHForward. It has to be pointed out that, due to practical restrictions related to the beamtime available and the parasitic run with other experiments, it was not possible to work on the optimization of the beam optics to achieve the nominal goal resolution for the slice emittance measurements, which is in the sub-10 fs range. Despite this limitation, those data-sets constitute the first slice emittance measurements in both transverse directions using the same RF-structure ever performed in the particle accelerator community. Such kinds of measurements have been now enabled by the PolariX TDS design and performances.

Quadrupole scans were used for the measurements covering a total phase advance of $0.45\cdot 2 \pi$ in the horizontal plane and $0.55 \cdot 2\pi$ in the vertical plane. A total of twelve different optics were applied for the horizontal slice emittance scan and seven for the vertical scan. Five images were taken for every setting. The TDS resolution was kept constant by not changing the quadrupoles between the TDS and the measurement screen. Unfortunately, the two measurements had to be performed on different days due to time constraints, therefore the two input bunches are different.

**Figure 4:**
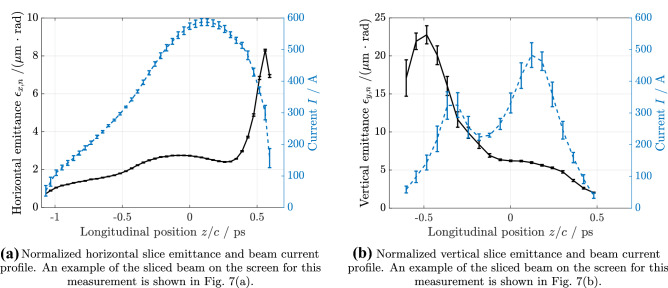
Normalized slice emittance. The errorbars indicate one standard deviation.

For the horizontal slice-emittance measurement the beam energy was $E={1120}\, \hbox {MeV}$, the rms bunch length was $\sigma _t\approx {400}\, {\mathrm{fs}}$, the charge was $Q={0.6}\, \hbox {nC}$, and the temporal TDS resolution was $R_t=(17\pm 1){\mathrm{fs}}$. Figure 4a displays the horizontal slice emittance analysis. The slice emittance in the core part of the bunch is in the range of 1 mm mrad to 3 mm mrad, c.f. Fig. 4a, as is expected for FLASHForward driver bunches^35^.

For the vertical slice emittance measurement the beam energy was $E={680}\, \hbox {MeV}$, the rms bunch length was $\sigma _t\approx {270}\, {\mathrm{fs}}$, the charge was $Q={0.3}\, \hbox {nC}$, and the temporal TDS resolution was $R_t=(56\pm 3){\mathrm{fs}}$. The vertical slice emittance analysis is shown in Fig. 4b. For this measurement, a second experiment was running in parallel, which made use of some advanced compression schemes. We tried to compensate these compression settings, but nevertheless the bunch obtained a double peak in the longitudinal profile. Also, the slice emittance increased from what we usually expect for FLASH driver bunches, as can be seen in Fig. 4b. This emittance increase especially in the tail of the bunch most likely stems from space charge and CSR effects in the FLASH bunch compressors and the FLASH2 and FLASHForward extraction.

### Three-Dimensional Bunch Charge Density Reconstruction

The theoretical concept of retrieving the three-dimensional charge density distribution of an electron bunch by using a tomographic algorithm to analyze images of the beam streaked by the PolariX TDS at multiple directions was numerically studied in previous works. In particular, for the technical description of the methodology for obtaining the beam charge density reconstruction we refer to^42^ and to “Algorithm for three-dimensional charge density reconstruction”. The reliability of the method used for the reconstruction was bench-marked using well-known particle tracking codes such as ASTRA and Elegant^43,44^ with simulation data. In this article we discuss the result of the first experimental measurements.

**Figure 5:**
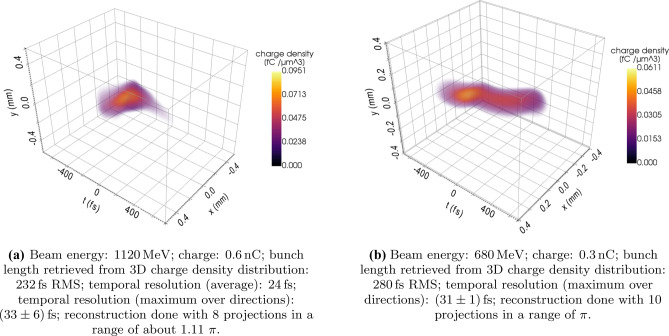
Three-dimensional charge density reconstruction. Reconstruction results from two different sets of experimental data. The 3D render was done with PyVista, which is a python implementation of VTK^45^. The normalization to the beam total charge value has been done after having set to 0 the voxels with negative values, constituting the halo of the figure and arising from noise of the reconstruction algorithm.

**Figure 6:**
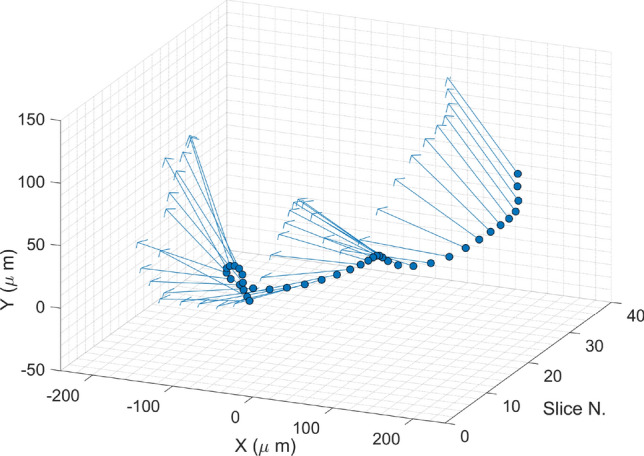
Centroids (dark blue points) and orientation of the x–y correlation (light blue arrows) in the longitudinal slices of the electron bunch calculated from the beam reconstructed in Fig. 5b. The slices have length of 31 fs and increasing running number scrolling from the head to the tail the beam distribution.

If the longitudinal bunch profile is constant between the TDS and the measurement screen, we can retrieve the three-dimensional charge-density distribution of the bunch at the location of the measurement screen.

During the collection of the data, the beam optics was not varied to prevent perturbation of the goal distribution at the screen location. The optics upstream of the TDS was optimized to have a nearly symmetric evolution of the betatron functions in x and y and a round minimum spot-size at the screen location. Moreover, in order to ensure the same streak parameter for all deflection angles, the quadrupoles between the TDS and the screen were kept off.

We collected data for the reconstruction during two shift-blocks characterized by different beam parameters. The choice of switching off the quadrupoles between the TDS and the measurement screen makes the longitudinal resolution of this measurement worse than the optimal one achievable for slice emittance measurements; indeed the phase advance between the TDS and the screen can not be independently optimised. Anyway in our measurements we were not limited by the resolution since the RMS bunch length was always a factor 7–10 longer.

In the first shift block, we characterized a beam with an energy of 1120 MeV, about ${0.6}\, \hbox {nC}$ charge and 230fs RMS bunch length. We collected 8 projections in a range of about 200 deg of the azimuth of the streaking (for more details see “Algorithm for three-dimensional charge density reconstruction”. The result of the reconstructed beam distribution is shown in Fig. 5a.

In the second shift block, we characterized a beam having an energy of 680 MeV, about ${0.3}\, \hbox {nC}$ charge and 270fs fs RMS bunch length (see Fig. 5b). We collected ten projections in a range of $\pi$. The principal features characterizing the bunch shape distribution, such as the double-bend in the longitudinal shape of the electron bunch can be diagnosed by collecting just a few streaked images. Nevertheless, the details of the two-dimensional transverse distribution of the longitudinal slices are influenced by the number of projections collected in the reconstruction (see Supplementary Fig. 1), as it is typically observed in tomography.

In Fig. 6 we show the position of the centroids and the angle of the correlation of the slice particle distribution in $x-y$ shown in Fig. 5b. A modulation in the angle of the correlation along the beam was detected.

### Discussion of the results

In this article, we presented the first characterization of the electron beam at FLASH performed using the PolariX TDS.

The FLASH facility at DESY is running since 2004 and many beam characterization methods have been developed to allow better understanding and optimization of the beam transport for user operation. Recently in^46^ it has been summarized as the quality of the FEL production for user increased proportionally with a research and development program covering the improvement of critical components of the beamline (e.g. the RF-gun), beam dynamics effects (compression to ultra-short bunches) and technical instrumentation (fs timing and synchronization).

Still remain aspects of the optimization of the beam quality that could be only partially optimized due to the complexity of the underlying physics and the limitations of the available diagnostics tools: CSR and wakefields induced by the RF-cavities as well as skewed perturbations caused by e.g. high order modes couplers remain subject of extensive study.

Coherent synchrotron radiation - coupled with space charge force - strongly influences the shape of the electron bunch after compression. In the CSR effect, the head of the beam is perturbed by the radiation emitted by the tail. Particles in the bunch radiate coherently at wavelengths much longer than the bunch length. This radiation produces a position-dependent energy modulation along the bunch, which will show up in the longitudinal phase space and distort it. A bunch distribution perturbed by CSR exhibits a shift of the centroids of the longitudinal slices of the electron bunch. Such effect could be studied so far using conventional transverse deflection structures and looking at slice centroids offsets in the plane perpendicular to the streaking direction of the structure^47^. Before the installation of the PolariX TDS the slice centroid offset in all transverse directions had never been detectable.

Although the exact benchmark of the measured beam shape with numerical codes is outside the scope of this article, we could observe shifts of the slice centroids at different directions both from the images of the streaked beam presented e.g. in Fig. 3 and in the three-dimensional beam charge density reconstruction shown in Fig.5(b) and 6.

In^27^, simulations of the 3D reconstruction measurement in preparation to the PolariX TDS commissioning at FLASHForward were performed. Certain features of the bunch shape experimentally obtained and shown in Fig 5(b) are qualitatively similar to what we expected from the previous study. The beam exhibits a modulation in the x-z plane that can be explained by the effect of CSR after the transit in the bunch compressors and the two extraction chicanes (all oriented in the horizontal direction). The measured offsets in the X-Y plane anyway as well as the longitudinal "beating" of the $x-y$ slice correlation term are more complex features that need further investigation to be better understood.

Possible sources of x-y coupling in the electron bunch are already present in the photo-injector region. In the PITZ facility, the RF-gun developed for FLASH are built and characterized. Transverse beam asymmetry was always observed from experiments looking at the transverse beam spot-size on a screen downstream the RF-gun^48^. A gun RF-coupler kick was found from RF -field simulations to be responsible for such shape^49^ and this effect has been recently mitigated introducing the use of a gun quadrupole^50^. Effects of transverse kicks from High Order Modes of the radio-frequency have been observed also looking at the beam trajectory in the RF-cavities constituting the FLASH linac^51^.

The three-dimensional bunch reconstruction method that we have experimentally demonstrated in this article allows to further investigate those aspects.

The inclusion of an X-band TDS with the ability to reconstruct the three-dimensional profile of particle bunches opens up new opportunities also in the context of beam-driven plasma accelerators. Firstly, longitudinal characterization of the beams driving the acceleration process allows for manipulation of key beam parameters. With such measurements the peak current of the driving beams could be maximized to increase the accelerating gradients with simultaneous manipulation of the longitudinal current profile used to optimize the efficiency of energy transfer in the system due to beam loading of the wakefields^2^. Secondly, characterization of the accelerated bunches provides key insight into the acceleration process itself. With this information experimental results can be used to benchmark state-of-the-art particle-in-cell numerical simulation packages, thus providing an iterative loop for deeper understanding of the underlying physics processes involved. Finally, due to the incredibly high accelerating and focusing gradients inherent to novel accelerator schemes, the fidelity of the acceleration process is extremely sensitive to particular bunch properties. For example, transverse offsets in the centroids of the beam can drive strong instabilities during the acceleration process^3^, leading to beam losses. Such a diagnostic device enables the mitigation of harmful beam properties.

Although multi-shot, the three-dimensional beam charge density reconstruction technique is extremely simple and, to the authors knowledge, it represents the first and only technique currently available for detailed characterization of the charge-density distribution of GeV-energy beams.

The possibility of detecting and monitoring the shape of the electron bunch, offset of the centroids of the slices and the rotation of the x-y correlation along the longitudinal coordinate of the beam distribution, that we have quantitatively shown to be possible, opens up new opportunities for automatised beam optimization using machine learning algorithm^52^. An adaptive tuning method has already been used for example at FACET to optimize beam transport in the linac while monitoring the image of the longitudinal phase space of the beam obtained by the combination of a conventional TDS and a dipole magnet^53^. We showed that the measurement of three-dimensional bunch density reconstruction can be performed with tens of fs longitudinal resolution and it is sufficiently robust with respect to shot-to-shot beam fluctuation. In the studied cases, we were able to characterize the electron beam with sufficient accuracy with a number of projections ranging from four to ten. Three-dimensional beam charge density reconstruction has therefore the potential to become a relatively quick measurement that can be integrated in automatized optimization algorithms.

In our studies, we were also able to confirm the possibility of slice emittance measurements in both transverse planes using the same deflecting structure. It is clear that for a proper characterization of the slice parameters of a beam, the measurements in both planes have to be performed using bunches of the same set up, ideally without much time separation. We will further investigate this in the near future. Also the optimization of the beam optics for reaching the nominal temporal resolution for the slice emittance measurements in the FLASHForward beamline will be performed in future studies.

The measurement of the slice emittance for arbitrary transverse planes is of extreme importance in the FEL community, since it enables a better control of the FEL transverse coherence properties. The possibility of performing such measurements with the same RF-structure has tremendous practical benefits in terms of compactness and costs of such beam diagnostic. It is worth to stress that for low energy particle-beams, where velocity bunching is not negligible^21,28^, the characterization of slice emittance on different planes using TDSs installed in series would be extremely complex. The beam indeed undergoes a quick evolution along the diagnostics drift which implies that integrating measurements obtained streaking the beam at different longitudinal position in the beamline is not trivial. Moreover the PolariX TDS design allows to extend the slice-emittance measurement to any arbitrary transverse direction, thus opening the path to six-dimensional beam phase space tomography, if the lattice in which the structure is installed can generate dispersion in multiple planes. In conclusion we expect it and the newly enabled beam characterization techniques that we have discussed to likely strongly influence the way of designing the diagnostics beamline for future accelerators and to allow better understanding of many aspects of beam dynamics and bunch quality optimization.

## Methods

In this Section we would like to provide more details concerning the experimental setup, the theory for the data analysis of the slice emittance measurement and the algorithm used for the three-dimensional charge density reconstruction. Finally we will comment on the sources of errors for the analyzed data.

### Experiment set-up

The beam distributions collected at the screen 11FLFXTDS were measured using a scintillation screen made of GAGG (gadolinium aluminium gallium garnet, Gd$_3$Al$_2$Ga$_3$O$_{12}$:Ce)^54^. The screen has a size of ${24.0}\, \hbox {mm}\times {30.5}\, \hbox {mm}$ and a thickness of ${200}\, \upmu \hbox {m}$. It is tilted by ${45}^{\circ }$ with respect to the beam direction and it is imaged into a CCD camera (model Basler aviator avA2300-25gm) using the Scheimpflug principle with a magnification ratio of 1:1. The size of each of the $1750 \times 2330$ pixels is ${5.4}\, \upmu \hbox {m}\times {5.6}\, \upmu \hbox {m}$ leading to a field of view of ${9.45}\, \hbox {mm}\times {13.05}\, \hbox {mm}$. The setup provides a spacial resolution of about 10 μm.

### Theory for slice emittance evaluation

The emittance is measured by observing $n\ge 3$ beam sizes with *n* different transfer matrices $\mathbf {M}^i_{s_1\leftarrow s_0}$ between the reconstruction point at $s_0$ and the screen at $s_1$, and $i\in \left[ 1,n\right]$ is the measurement index^55–57^. The indices $\left\{ q,p \right\}$ are $\left\{ 1,2 \right\}$ for the horizontal plane and $\left\{ 3,4 \right\}$ for the vertical plane, and $M_{qp}$ is the *q*, *p* element of $\mathbf {M}_{s_1\leftarrow s_0}$. With a vanishing $M_{q6}$ element the beam size at the screen can be calculated as

5 $$\begin{aligned} \left\langle v^2(s_1)\right\rangle = \sigma _{v}^2(s_1) = M_{qq}^2 \left\langle v^2(s_0) \right\rangle + 2 M_{qq} M_{qp} \left\langle v(s_0) v'(s_0) \right\rangle + M_{qp}^2 \left\langle v'^2(s_0) \right\rangle . \end{aligned}$$

By observing $n\ge 3$ beam sizes it is possible to determine $\left\langle v^2(s_0) \right\rangle$, $\left\langle v(s_0) v'(s_0) \right\rangle$, and $\left\langle v'^2(s_0) \right\rangle$ and calculate the emittance $\epsilon _v$ and the Twiss functions $\beta _v$ and $\alpha _v$ via^55–57^

6 $$\begin{aligned} \epsilon _v= & {} \sqrt{\left\langle v^2(s_0) \right\rangle \left\langle v'^2(s_0) \right\rangle -\left\langle v(s_0) v'(s_0) \right\rangle }, \end{aligned}$$

7 $$\begin{aligned} \beta _v(s_0)= & {} \frac{\left\langle v^2(s_0) \right\rangle }{\epsilon _v}, \end{aligned}$$

8 $$\begin{aligned} \alpha _v(s_0)= & {} -\frac{\left\langle v(s_0) v'(s_0) \right\rangle }{\epsilon _v}. \end{aligned}$$

Additionally, the mismatch between the design and the measured beam ellipse can be expressed by the mismatch parameter or beta matching parameter $B_\text {mag}$^57,58^

9 $$\begin{aligned} B_\text {mag}= \frac{1}{2} \left[ \beta _v(s_0)\gamma _{v_0}(s_0) -2\alpha _v(s_0) \alpha _{v_o}(s_0) +\beta _{v_0}(s_0)\gamma _v(s_0) \right] , \end{aligned}$$

where $\beta _v(s_0)$, $\alpha _v(s_0)$, and $\gamma _v(s_0)$ are the measured Twiss parameters, and $\beta _{v_0}(s_0)$, $\alpha _{v_0}(s_0)$, and $\gamma _{v_0}(s_0)$ are the design parameters. For a rigorous treatment on how to calculate the standard deviation for this measurement, we refer the reader to^55–57^.

Finally, the slice emittance can be determined, by streaking the beam in the *u* plane using the TDS^59^. For each measurement *i* the beam image is then sliced into *m* slices along the *u* axis. For each of the *m* slices the corresponding beam size $\left\langle \left[ v_m^i(s_2)\right] ^2 \right\rangle$ is measured *m* times (once for each slice). This yields the *m* slice emittances $\epsilon _{v,j}$, where $j\in \left[ 1,m\right]$.

**Figure 7:**
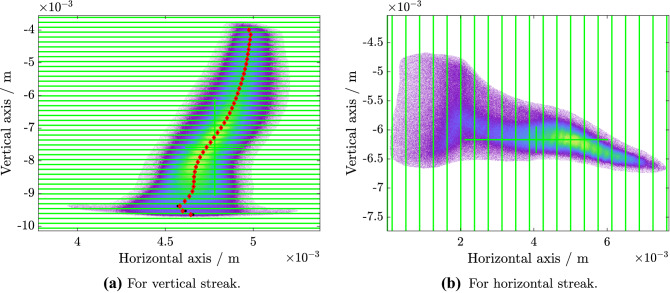
Sample sliced beam images on screen. The beam is streaked in the given direction by the PolariX TDS. For the analysis, the beam is divided into slices (green lines) around the centroid (green cross). The centroid positions within each slice are calculated using the rms method (black stars) and the center of a Gaussian fit (red diamonds).

Two sample images for the horizontal and vertical slice emittance analysis are displayed in Fig. 7.

**Figure 8:**
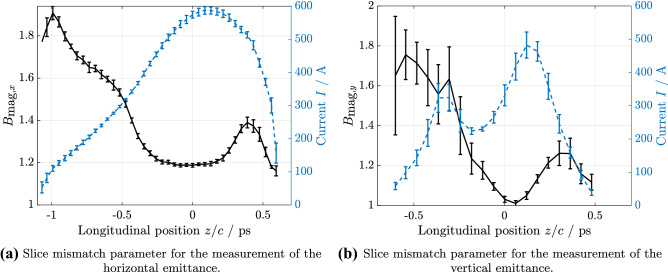
Slice mismatch parameters for the emittance measurements.

From Fig. 8 it is evident, that the beam was not perfectly matched to the theory optics at this position.

### Algorithm for three-dimensional charge density reconstruction

**Figure 9:**
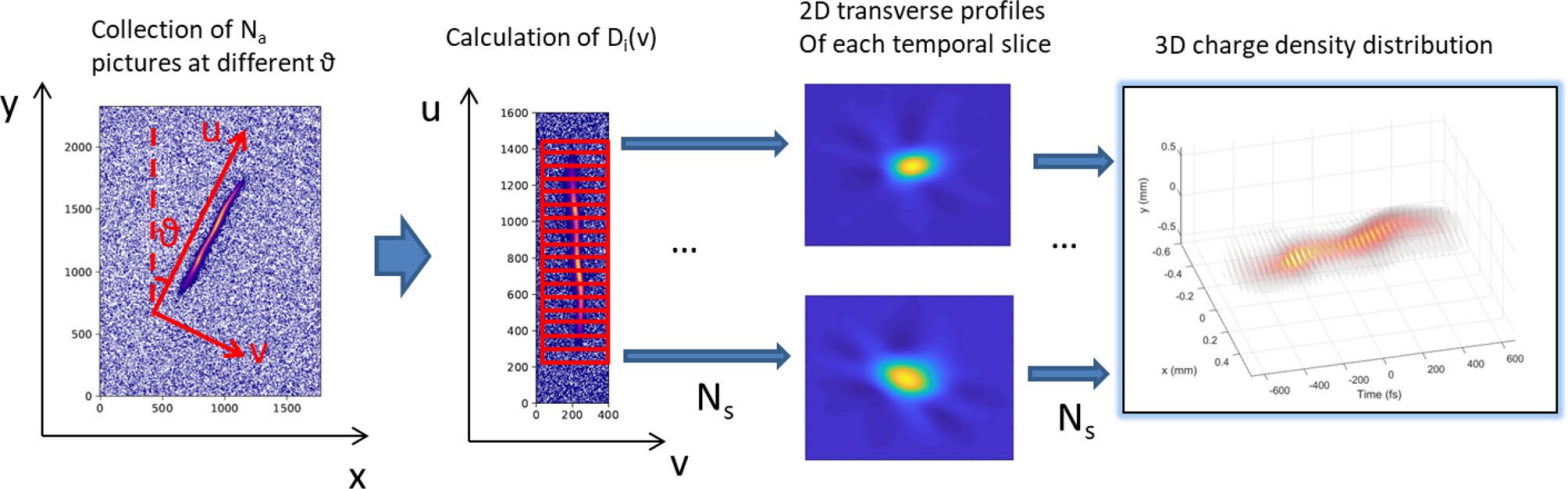
Algorithm for the tomographic reconstruction. The scheme illustrates schematically the conceptual steps performed in the data analysis of the images of the streaked beam to obtain a 3D reconstruction of the charge density distribution of the bunch.

The general procedure for the measurement is illustrated in Fig. 9.

- First of all, images of the streaked beam at $N_a$ different streaking directions are taken. To minimize the artifacts introduced by the reconstruction algorithm, the chosen angles should be roughly equally spaced and should cover a range of $\pi$.
- The angle of the projection $\theta$ is identified during the calibration procedure. Each image is analyzed in the $u-v$ plane which is defined as illustrated in the left side of Fig. 9. The time-profile of each image is divided in $N_s$ longitudinal slices, whose length must be bigger than the longitudinal-resolution $R_z$. The transverse distributions $D_{i,\theta }(v)$ of each longitudinal slice *i* for an angle $\theta$ are collected.
- For each longitudinal slice *i*, the $N_a$ one-dimensional-profiles $D_{i,\theta }(v)$ are combined using a tomographic reconstruction algorithm to obtain a two-dimensional-slice profile. In this work the well-known Simultaneous Algebraic Reconstruction Technique (SART)^60^ using the Kaczmarz iterative solver method^61^, has been applied with two iterations, using the implementation in the scikit-image package^62^.
- Finally the two-dimensional slices are stacked together to form a complete three-dimensional charge profile reconstruction. Those slices are interpolated with a three-dimensional rendering done with PyVista, a python implementation of VTK^45^.

For the measurements presented in this article, we have collected five images for each streaking angle. Only one image per streaking angle is used for the reconstruction of the three-dimensional charge density distribution. Nevertheless, we have compared reconstructed distributions using different shots for the selected angles in order to confirm that the reconstruction is not affected by shot-to-shot fluctuations (see Supplementary Fig. 2).

For the beam reconstruction shown in Fig. 5 we used the average value of the streak parameter measured for different directions. More details concerning the precision and homogeneity of the calibration at different angles are going to be presented in in a separate article currently under preparation^41^.

We have investigated the differences in the reconstructed image with respect to the number of angles scanned. We have also verified the impact of the inclusion in the measurement of both zero crossings of the RF-phase of the TDS. It is worth nothing that for the PolariX TDS the measurement at two zero crossings of the RF-phase is equivalent to the measurement at the same RF-phase but with a change of $\pi$ of the azimuth of the streaking. In both cases indeed the direction of the streaking observed on the measurement screen is not changed but the versor of the streaking of the head of the beam is flipped. For this reason, taking measurements at both zero crossings of the RF-phase is equivalent to spanning 2$\pi$ range in the collection of the beam projections instead of $\pi$. In absence of imperfections in the RF fields or higher order effects in the beam dynamics during the transit in the structure, this extension of the angle range should not be necessary.

The data analyzed confirmed that for the bunch under observation the optimization of the longitudinal resolution of the measurement played a more important role with respect to the collection of a high number of projections and the extension of the phase range to 2$\pi$ (see Supplementary Fig. 3).

### Sources of errors

The main errors in the presented measurements are governed by statistical errors in the multi-shot calibration. The arrival time jitter of the beam and the RF-phase jitter of the TDS are of the order of tens of fs and constitute the main source of noise in the calibration procedure. The errors presented in the article are calculated by applying error propagation rules to the initial statistical errors.

The streak parameter used for the tomographic reconstruction is given by the average of the values measured at different azimuth. The uncertainty on the measured streak parameter represents one of the dominant errors of the reconstruction.

Another systematic error is caused by artifacts introduced in the reconstruction algorithm.

To have an estimation of the impact of such artifacts we have compared the reconstructed beam distribution projected on the transverse plane (*X*, *Y*) (shown in Supplementary Figs. 1 and 3) with the measured spot-size of the beam when the PolariX TDS was switched OFF (see Supplementary Fig. 4).

Moreover in the caption of Supplementary Fig. 1 we have compared the RMS bunch length values measured in the following situations:

- From the average length of the temporal profile of two streaked images at opposite azimuths (this is the conventional method used for TDSs). In this case the streak parameter measured at those specific streaking angle were considered.
- Using the projected distribution from the tomographic reconstruction with different numbers of projections (4, 10 and 20). In those cases the average streak parameter measured considering 20 projections has been considered.

The 4 measured values agree within the errorbar determined by the first measurement and overall differ by less than 5 %.

## Supplementary Information

Supplementary Movie 1.

Supplementary Information

## Data Availability

The datasets generated during and/or analyzed during the current study are available from the corresponding author on reasonable request.
