# Supplementary figures and images for: Experimental demonstration of novel beam characterization using a polarizable X-band transverse deflection structure
Source: Sci Rep. 2021 Feb 11;11:3560. doi: 10.1038/s41598-021-82687-2 (PMC7878911; doi:10.1038/s41598-021-82687-2)

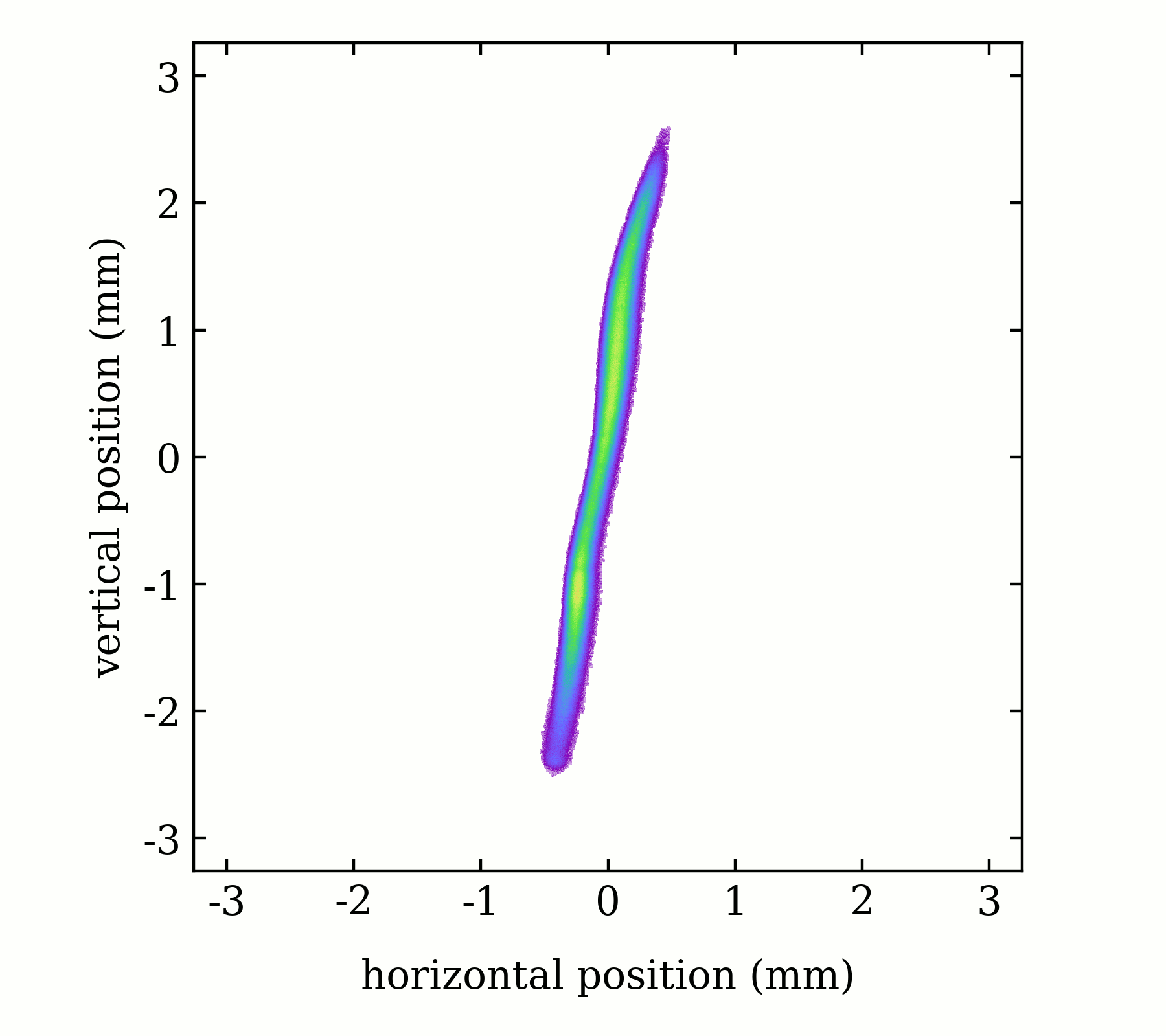

Supplement: Supplementary file 1 — Supplementary Movie 1. [file 41598_2021_82687_MOESM1_ESM.gif]
