# Supplementary material for: Experimental demonstration of novel beam characterization using a polarizable X-band transverse deflection structure
Source: Sci Rep. 2021 Feb 11;11:3560. doi: 10.1038/s41598-021-82687-2 (PMC7878911; doi:10.1038/s41598-021-82687-2)
Supplement: Supplementary file 2 — Supplementary Information [file 41598_2021_82687_MOESM2_ESM.docx]

**Experimental Demonstration of novel Beam Characterization using a PolarizableX-Band Transverse Deflection Structure**

B. Marchetti^*^, R. Assmann, F. Christie, R. D’Arcy, P. González Caminal, M. Hoffmann, M. Huening, S. M. Jaster-Merz, R. Jonas, D. Marx^†^, J. Osterhoff, M. Reukauff, S. Schreiber, G. Tews, M. Vogt, and S. Wesch

Deutsches Elektronen-Synchrotron, 22607 Hamburg, Germany

A. Grudiev^‡^, N. Catalan Lasheras, G. Mcmonagle and W. Wuensch

CERN, 1211 Geneva 23, Switzerland

P. Craievich^§^, H. Braun, R. Fortunati, R. Ganter, F. Marcellini, M. Pedrozzi, E. Prat and S. Reiche

PSI, 5232 Villigen, Switzerland

_____________________________________________________________________

^*^ barbara.marchetti@desy.de; Present address: European XFEL Company, Holzkoppel 4, 22869 Schenefeld, Germany

^†^ Present address: Brookhaven National Laboratory, Upton, NY11973-5000, USA

^‡^ Alexej.Grudiev@cern.ch

^§^ paolo.craievich@psi.ch


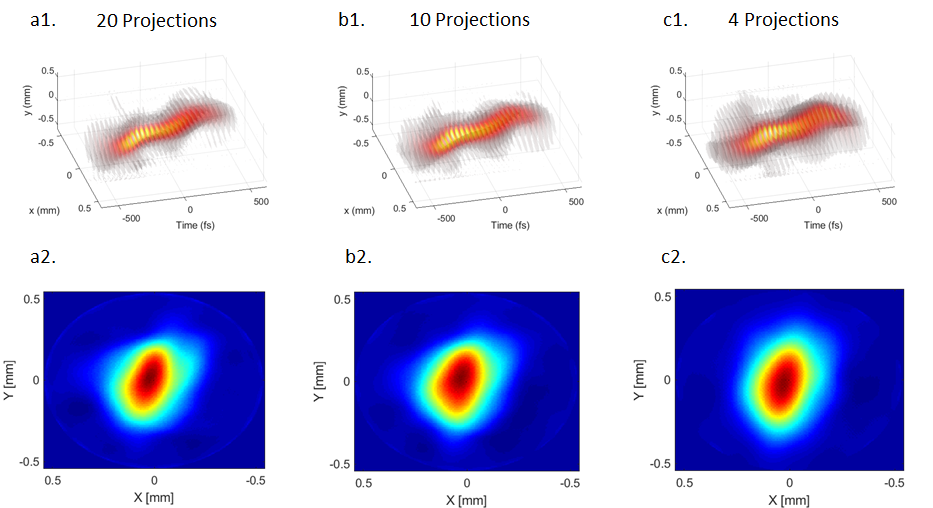


Supplementary Figure 1: Reconstructed beam distribution using a different number of projections over 360 deg range. Beam energy= 680 MeV, bunch charge=0.3nC, bunch length = (261±28) fs RMS, longitudinal resolution (maximum value) = (36±3) fs. The panels a1.-b1.-c1. show the reconstructed 3D charge density distribution from 20, 10 and 4 initial angles of the streaking respectively. The calculated RMS bunch length values for the 3 cases are: 262 fs, 268 fs and 273 fs respectively. The panels a2.-b2.-c2. show the reconstruction of the projected transverse distribution of the beam.


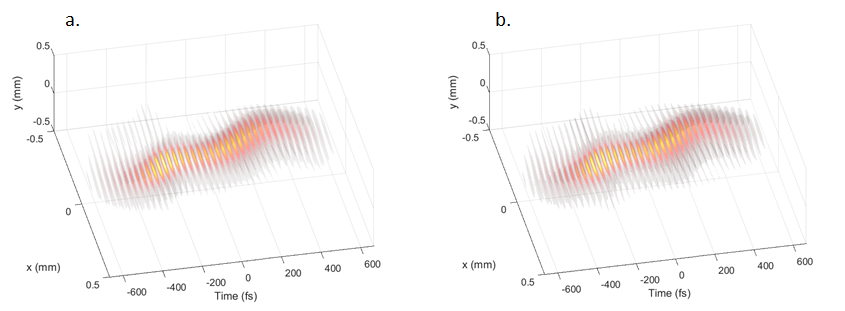


Supplementary Figure 2: Shot-to-shot stability of the reconstruction. Panels a. and b. show the 3D charge-density reconstruction for the working point presented in Fig. 5.b of the main article using different initial sets of 10 shots of the streaked beam for a fixed set of angles of the projection.


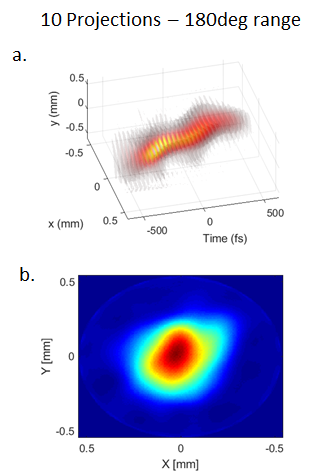


Supplementary Figure 3: Influence of the zero-crossings of the RF-phase. Analysis of the same data used for Supplementary Figure 1. Panel a. shows the reconstruction of 3D beam charge-density distribution using 10 projections distributed over 180 deg. Panel b. shows the reconstructed projection of the transverse beam distribution for the same case. The quality of the reconstruction is similar to the one obtained for 10 projections over 360 deg range, shown in Supplementary Figure 1.


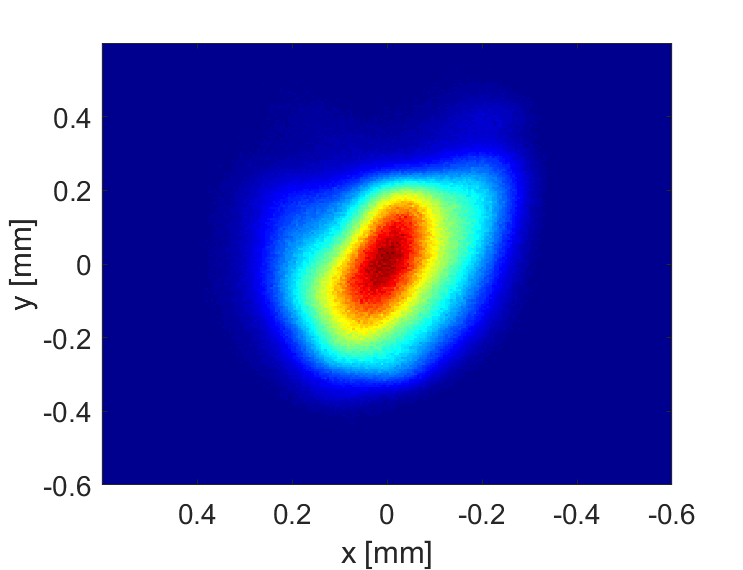


Supplementary Figure 4: Measured spot-size at 11FLFXTDS with PolariX TDS switched OFF. This distribution serves as a benchmark for the reconstructed transverse distributions presented in Supplementary Fig. 1 and 3.
